# Random Weighting, Strong Tracking, and Unscented Kalman Filter for Soft Tissue Characterization

**DOI:** 10.3390/s18051650

**Published:** 2018-05-21

**Authors:** Jaehyun Shin, Yongmin Zhong, Denny Oetomo, Chengfan Gu

**Affiliations:** 1 School of Engineering, RMIT University, Bundoora, VIC 3083, Australia; yongmin.zhong@rmit.edu.au; 2 Department of Mechanical Engineering, University of Melbourne, Parkville, VIC 3010, Australia; doetomo@unimelb.edu.au; 3 City of Whittlesea, Mill Park, VIC 3082, Australia; Charly.Xie1@yahoo.com or chengfan.gu@rmit.edu.au

**Keywords:** soft tissue characterization, Hunt-Crossley model, unscented Kalman filter, contact model error, strong tracking, and random weighting

## Abstract

This paper presents a new nonlinear filtering method based on the Hunt-Crossley model for online nonlinear soft tissue characterization. This method overcomes the problem of performance degradation in the unscented Kalman filter due to contact model error. It adopts the concept of Mahalanobis distance to identify contact model error, and further incorporates a scaling factor in predicted state covariance to compensate identified model error. This scaling factor is determined according to the principle of innovation orthogonality to avoid the cumbersome computation of Jacobian matrix, where the random weighting concept is adopted to improve the estimation accuracy of innovation covariance. A master-slave robotic indentation system is developed to validate the performance of the proposed method. Simulation and experimental results as well as comparison analyses demonstrate that the efficacy of the proposed method for online characterization of soft tissue parameters in the presence of contact model error.

## 1. Introduction

Soft tissue properties are important for robotic-assisted minimally invasive surgery to achieve realistic haptic feedback and stable robotic control. However, soft tissue properties are dynamically changing depending on tissue layers, organs, patients, and physiological conditions. Accordingly, it is necessary to online characterize dynamic soft tissue properties from tool-tissue interaction measurements for robotic-assisted minimally invasive surgery [1,2,3].

The identification of soft tissue properties relies on the knowledge of the contact interaction between surgical tool and soft tissues. Due to the computational efficiency, various contact models have been used to describe the mechanical contact with soft tissues. The simple one is the linear elastic model, which fails to represent the complex behaviour of tool–tissue interaction [4]. The Maxwell (MW) model is constructed by a spring and damper in serial form [5], while the Kelvin–Voigt (KV) model a spring and damper in parallel form [6]. The Kelvin–Boltzmann (KB) model adds a spring in serial form to the Kelvin–Voigt model [7]. However, these three are linear spring-damper models and involves unnatural forces at start and end points of the contact [8]. The Hunt-Crossley (H-C) model is nonlinear and solves the unnatural force problem involved in the linear K-V model [8,9]. Its nonlinearity is also suitable for characterization of nonlinear soft tissue behaviours for robotic-assisted minimally invasive surgery. However, the use of the nonlinear H-C model for online soft tissue characterization requires an online nonlinear estimation algorithm, which is more complicated than an online linear estimation algorithm. Thus, there has been limited research on using the nonlinear H-C model for soft tissue characterization. Although finite element model (FEM) [10] provides an accurate description of the force interaction between surgical tool and soft tissues using continuum mechanics, it is difficult to achieve the real-time performance due to expensive computations [3,11].

In addition to a dynamic contact model, an online estimation algorithm is also required for real-time soft tissue characterization. The recursive least square (RLS) is an online estimation algorithm for soft tissue characterization [8,9]. However, its performance is sensitive to initial conditions [9]. The Kalman filter (KF) is an improvement to RLS. It can provide estimations in accuracy of minimum mean-square error. However, both RLS and KF are a linear estimation algorithm, unsuitable for the use with the nonlinear H-C model for soft tissue characterization [8].

Various nonlinear versions of KF were reported by extending the concept of KF to nonlinear systems. The extended Kalman filter (EKF) is a commonly used nonlinear estimation algorithm. However, due to the involvement of model linearization, it can only achieve first-order accuracy [12,13]. EKF also requires the cumbersome computation of Jacobian matrix at each time point. The unscented Kalman filter (UKF) improves EKF by approximating state mean and covariance via unscented transformation [10,14]. It can achieve third-order accuracy. Further, it does not require the cumbersome calculation of Jacobian matrix. However, UKF requires an accurate system model. In robotic-assisted minimally invasive surgery, the system model always involve uncertainties such as inappropriate initial conditions, modelling error due to model simplification for the purpose of computational efficiency, unexpected system noises and stochastic drifts, leading to the deteriorated UKF solution [15,16]. Currently, there has been limited research focusing on the use of UKF for nonlinear soft tissue characterization. Xi et al. reported a method by combining UKF with a FEM to estimate myocardial material parameters [10]. However, the FEM is under the quasi-static assumption, which does not hold for a dynamic contact system. Further, the UKF estimation performance is degraded due to the reduced size for state vector’s error covariance. Recently, the authors also studied a method by combing UKF with the H-C model for soft tissue characterization [17]. However, the UKF problem in requirement of accurate system model was not addressed.

Adaptive filtering is a strategy to handle the disturbance of system model error on state estimation. It has been used with UKF, leading to the adaptive UKF to inhibit the influence of system model error on the filtering solution [18]. The innovation based adaptive estimation and residual based adaptive estimation are two typical methods for adaptive filtering [19], where the innovation or residual at present time point is adaptively estimated from all historical innovations or residuals within a small time window [20]. However, due to the use of equal weighting for all historical innovations within the time window, each historical innovation or residual within the time window equally contributes to the current innovation or residual, leading to limited estimation accuracy. The random weighting method overcomes this problem by taking into account different precision levels of historical innovations or residuals in the current innovation or residual via random weights, leading to improved estimation accuracy [15,21].

The strong tracking (ST) is a relatively new concept in adaptive filtering. It incorporates a scaling factor into predicted state covariance to compensate system model error [22,23,24]. In addition to the strong robustness against system model error, ST is also able to online track system state. However, ST requires the cumbersome evaluations of Jacobian matrix to calculate the scaling factor, leading to an extra computational burden.

This paper presents a new nonlinear filtering method based on the nonlinear H-C contact model by combining the concepts of ST and random weighting into UKF for online soft tissue characterization. This method adopts the ST concept to address the UKF problem of performance degradation due to contact model error. It identifies contact model error using the Mahalanobis distance. Subsequently, a scaling factor is introduced into predicted state covariance to account for identified contact model error. To avoid the cumbersome calculation of Jacobian matrix, this scaling factor is determined according to the orthogonality principle, where the random weighting concept is adopted to enhance the estimation accuracy of innovation covariance. Simulations, practical experiments, and comparison analysis with UKF have been conducted to comprehensively evaluate the performance of the proposed method.

## 2. Nonlinear Hunt-Crossley Contact Model

The nonlinear H-C contact model describes the dynamics of the contact between surgical tool and soft tissues via the following nonlinear equation [25]:

(1) $$F=Kd^{n}+Bd^{n}{\dot{d}}^{p}$$

where *F*, *K*, *B*, d, $\dot{d}$, *n*, and *p* are the contact force, stiffness coefficient, damping coefficient, displacement, displacement velocity, power of displacement, and power of displacement velocity, respectively.

Define the state vector as:

(2) $$x_{k}=\left[d{\left(t\right)}_{k},\;\dot{d}{\left(t\right)}_{k},\;F_{k},\;K_{k},\;B_{k},\;n_{k},\;p_{k}\;\right].$$

Based on Equation (1), the system state equation can be formulated as:

(3) $$x_{k}=f\left(x_{k-1}\right)+q_{k-1}=\left\{\begin{array}{c} d_{k-1}+{\dot{d}}_{k-1}\times \Delta t_{k-1}\\ {\dot{d}}_{k-1}\\ K_{k-1}d_{k-1}^{n_{k-1}}+B_{k-1}d_{k-1}^{n_{k-1}}{\dot{d}}_{k-1}^{p_{k-1}}\\ K_{k-1}\\ B_{k-1}\\ n_{k-1}\\ p_{k-1} \end{array}\right\}+q_{k-1}$$

where $x_{k}=\left[d{\left(t\right)}_{k},\;\dot{d}{\left(t\right)}_{k},\;F_{k},\;K_{k},\;B_{k},\;n_{k},\;p_{k}\;\right]$ is the system state at time point $t_{k}$, f(·) is the system function, and $q_{k}\sim \left(0,Q_{k}\right)$ is a white Gaussian noise with zero mean and covariance $Q_{k}$.

The measurement equation is defined as:

(4) $$y_{k}=h\left(x_{k}\right)+r_{k}=\left(\begin{array}{c} d_{k}\\ F_{k} \end{array}\right)+r_{k}$$

where $y_{k}$ is the measurement at time point $t_{k}$, *h*(·) is the measurement function which describes the relation between the measurement and system state, and $r_{k}\sim \left(0,R_{k}\right)$ is a white Gaussian noise with zero mean and covariance $R_{k}$.

It should be noted that in robotic-assisted minimally invasive surgery, the accuracy of the measurement model can be guaranteed, since force data can be obtained from a high-accurate force sensor and displacement data can be obtained from precise robotic encoder.

## 3. Analysis of Unscented Kalman Filter

The conventional UKF procedure can be described as follows:

**Step 1.** Initialization:

(5) $${\hat{x}}_{0}=E\left(x_{0}\right)$$

(6) $${\hat{P}}_{0}\;=E\left[\left(x_{0}-{\hat{x}}_{0}\right){\left(x_{0}-{\hat{x}}_{0}\right)}^{T}\right].$$

**Step 2.** Time update with unscented transformation:

Select sigma points:

(7) $${\hat{x}}_{k-1}^{\left(i\right)}={\hat{x}}_{k-1}+{\hat{x}}^{\left(i\right)}\;\left(i=1,\;\cdots ,\;2N\right)\;{\hat{x}}^{\left(i\right)}={\left(\sqrt[{}]{\left(N+\lambda \right){\hat{P}}_{k-1}}\right)}_{i}^{T}\;\left(i=1,\;\cdots ,\;N\right)\;{\hat{x}}^{\left(N+i\right)}=-{\left(\sqrt[{}]{\left(N+\lambda \right){\hat{P}}_{k-1}}\right)}_{i}^{T}\;\left(i=1,\;\cdots ,\;N\right)$$

where N is the dimension of state vector x, the parameter λ is defined as $\lambda =\alpha^{2}\left(N+k\right)-N$ with constant α, and ${\hat{P}}_{k-1}$ is the estimated state covariance at time point $t_{k-1}$ (*k* = 1, 2, …).

Calculate predicted state vector ${\bar{x}}_{k}\;$ based on the selected sigma points:

(8) $${\bar{x}}_{k}^{\left(i\right)}=f\left({\hat{x}}_{k-1}^{\left(i\right)}\right)\;{\bar{x}}_{k}=\frac{1}{2N}\overset{2N}{\underset{i=1}{\sum }}w_{i}^{m}\;\left({\bar{x}}_{k}^{\left(i\right)}\right).$$

Calculate predicted state covariance ${\bar{P}}_{k}$:

(9) $${\bar{P}}_{k}=\overset{2N}{\underset{i=1}{\sum }}w_{i}^{c}\left({\bar{x}}_{k}^{\left(i\right)}-{\bar{x}}_{k}\;\right){\left({\bar{x}}_{k}^{\left(i\right)}-{\bar{x}}_{k}\right)}^{T}+Q_{k}\;\begin{array}{l} w_{0}^{m}=\frac{\lambda }{n+\lambda }\\ w_{0}^{c}=\frac{\lambda }{n+\lambda }+\left(1-\alpha^{2}+\beta \right)\\ w_{i}^{m}=w_{i}^{c}=\frac{1}{2\left(N+\lambda \right)}\;i=1,\ldots ,2N \end{array}$$

where $w_{i}^{m}$ and $w_{i}^{c}$ are the mean and covariance weights, N is the dimension of state vector x, and α and β are constants.

**Step 3.** Measurement update:

Calculate predicted measurement ${\bar{y}}_{k}$:

(10) $${\bar{y}}_{k}^{\left(i\right)}=h\left({\bar{x}}_{k}^{\left(i\right)}\right)\;{\bar{y}}_{k}=\frac{1}{2N}\overset{2N}{\underset{i=1}{\sum }}w_{i}^{m}\left({\bar{y}}_{k}^{\left(i\right)}\right)$$

Calculate predicted measurement covariance $P_{{\bar{y}}_{k}}$:

(11) $$P_{{\bar{y}}_{k}}=\overset{2N}{\underset{i=1}{\sum }}w_{i}^{c}\left({\bar{y}}_{k}^{\left(i\right)}-{\bar{y}}_{k}\;\right){\left({\bar{y}}_{k}^{\left(i\right)}-{\bar{y}}_{k}\right)}^{T}+R_{k}$$

Calculate the cross covariance between ${\bar{x}}_{k}$ and ${\bar{y}}_{k}$:

(12) $$P_{{\bar{x}}_{k}{\bar{y}}_{k}}=\overset{2N}{\underset{i=1}{\sum }}w_{i}^{c}\left({\bar{x}}_{k}^{\left(i\right)}-{\bar{x}}_{k}\right){\left({\bar{y}}_{k}^{\left(i\right)}-{\bar{y}}_{k}\right)}^{T}$$

Calculate the Kalman gain:

(13) $$K_{k}=P_{{\bar{x}}_{k}{\bar{y}}_{k}}P_{{\bar{y}}_{k}}^{-1}$$

Update the estimated state and associated covariance:

(14) $${\hat{x}}_{k}={\bar{x}}_{k}+K_{k}Z_{k}^{I}$$

(15) $${\hat{P}}_{k}={\bar{P}}_{k}-K_{k}P_{{\bar{y}}_{k}}K_{k}^{T}$$

where $Z_{k}^{I}=y_{k}-{\bar{y}}_{k}$ is called the innovation vector.

Now let us analyse the effect of contact model error on the UKF performance. The predicted state in Equation (8) can be rewritten as:

(16) $${\bar{x}}_{k}=\frac{1}{2N}\overset{2N}{\underset{i=1}{\sum }}w_{i}^{m}\;\left(f\left({\bar{x}}_{k-1}^{\left(i\right)}\right)\right).$$

Since the contact state Equation (3) involves error $f_{e}\left(\cdot \right)$, state prediction error ${\overset{ˇ}{x}}_{k}$ can be represented as:

(17) $${\overset{ˇ}{x}}_{k}=\frac{1}{2N}\overset{2N}{\underset{i=1}{\sum }}w_{i}^{m}\;f_{e}\left({\bar{x}}_{k-1}^{\left(i\right)}\right).$$

The predicted state in the presence of model error $f_{e}\left(\cdot \right)$ can be represented as:

(18) $$\overset{ˇ}{\bar{x}}={\bar{x}}_{k}+{\overset{ˇ}{x}}_{k}=\frac{1}{2N}\overset{2N}{\underset{i=1}{\sum }}w_{i}^{m}\;\left(f\left({\bar{x}}_{k-1}^{\left(i\right)}\right)\right)+\frac{1}{2N}\overset{2N}{\underset{i=1}{\sum }}w_{i}^{m}\;f_{e}\left({\bar{x}}_{k-1}^{\left(i\right)}\right).$$

Using unscented transformation, the predicted state covariance in the presence of model error $f_{e}\left(\cdot \right)$ can be calculated as:

(19) $${\overset{ˇ}{\bar{P}}}_{k}=\overset{2N}{\underset{i=1}{\sum }}w_{i}^{c}\left({\bar{x}}_{k}^{\left(i\right)}-{\bar{x}}_{k}+{\overset{ˇ}{x}}_{k}^{\left(i\right)}-{\overset{ˇ}{x}}_{k}\right){\left({\bar{x}}_{k}^{\left(i\right)}-{\bar{x}}_{k}+{\overset{ˇ}{x}}_{k}^{\left(i\right)}-{\overset{ˇ}{x}}_{k}\right)}^{T}+Q_{k}.$$

where ${\overset{ˇ}{x}}_{k}^{\left(i\right)}$ and ${\bar{x}}_{k}^{\left(i\right)}$ are the sigma points selected from ${\overset{ˇ}{x}}_{k}$ and ${\bar{x}}_{k}$, respectively.

Denote:

(20) $${\bar{X}}_{k}^{\left(i\right)}={\bar{x}}_{k}^{\left(i\right)}-{\bar{x}}_{k}\;{\overset{ˇ}{X}}_{k}^{\left(i\right)}={\overset{ˇ}{x}}_{k}^{\left(i\right)}-{\overset{ˇ}{x}}_{k}.$$

Thus, Equation (19) can be further written as:

(21) $${\overset{ˇ}{\bar{P}}}_{k}=\overset{2N}{\underset{i=1}{\sum }}w_{i}^{c}\left({\bar{X}}_{k}^{\left(i\right)}+{\overset{ˇ}{X}}_{k}^{\left(i\right)}\right){\left({\bar{X}}_{k}^{\left(i\right)}+{\overset{ˇ}{X}}_{k}^{\left(i\right)}\right)}^{T}+Q_{k}\;=\overset{2N}{\underset{i=1}{\sum }}w_{i}^{c}\left({\bar{X}}_{k}^{\left(i\right)}{\bar{X}}_{k}^{\left(i\right)}{}^{T}+{\bar{X}}_{k}^{\left(i\right)}{\overset{ˇ}{X}}_{k}^{\left(i\right)}+{\overset{ˇ}{X}}_{k}^{\left(i\right)}{\bar{X}}_{k}^{\left(i\right)}{}^{T}+{\overset{ˇ}{X}}_{k}^{\left(i\right)}{\overset{ˇ}{X}}_{k}^{\left(i\right)}{}^{T}\right)+Q_{k}.$$

Define the error ${\overset{ˇ}{P}}_{k}$ of predicted state covariance:

(22) $${\overset{ˇ}{P}}_{k}=\overset{2N}{\underset{i=1}{\sum }}w_{i}^{c}\left({\bar{X}}_{k}^{\left(i\right)}{\overset{ˇ}{X}}_{k}^{\left(i\right)}+{\overset{ˇ}{X}}_{k}^{\left(i\right)}{\bar{X}}_{k}^{\left(i\right)}{}^{T}+{\overset{ˇ}{X}}_{k}^{\left(i\right)}{\overset{ˇ}{X}}_{k}^{\left(i\right)}{}^{T}\right).$$

Substituting Equation (20) into Equation (21) yields:

(23) $${\bar{P}}_{k}=\overset{2N}{\underset{i=1}{\sum }}w_{i}^{c}\left({\bar{X}}_{k}^{\left(i\right)}{\bar{X}}_{k}^{\left(i\right)}{}^{T}\right)+Q_{k}.$$

Considering Equations (22) and (23), Equation (21) can be further written as:

(24) $$\begin{array}{l} {\overset{ˇ}{\bar{P}}}_{k}=\overset{2N}{\underset{i=1}{\sum }}w_{i}^{c}\left({\bar{X}}_{k}^{\left(i\right)}{\bar{X}}_{k}^{\left(i\right)}{}^{T}\right)+Q_{k}+\overset{2N}{\underset{i=1}{\sum }}w_{i}^{c}\left({\bar{X}}_{k}^{\left(i\right)}{\overset{ˇ}{X}}_{k}^{\left(i\right)}+{\overset{ˇ}{X}}_{k}^{\left(i\right)}{\bar{X}}_{k}^{\left(i\right)}{}^{T}+{\overset{ˇ}{X}}_{k}^{\left(i\right)}{\overset{ˇ}{X}}_{k}^{\left(i\right)}{}^{T}\right)\\ ={\bar{P}}_{k}+{\overset{ˇ}{P}}_{k}. \end{array}$$

It can be seen from Equation (24) that model error $f_{e}\left(\cdot \right)$ causes predicted state covariance’s error ${\overset{ˇ}{P}}_{k}$, leading to the inaccurate Kalman gain. Therefore, the state estimate will be degraded when the contact model involves error.

## 4. Random Weighting Strong Tracking Unscented Kalman Filter

This paper presents a random weighting strong tracking unscented Kalman filter (RWSTUKF) to address the UKF problem of performance degradation in the presence of model error for nonlinear soft tissue characterization. This method corrects predicted state covariance using the ST concept to restrain the disturbance of model error on state estimation, and also adopts the random weighting concept to improve the estimation accuracy of innovation covariance.

### 4.1. Correction of Predicted State Covariance

As analysed above, the predicted state covariance has the deviation ${\overset{ˇ}{P}}_{k}$ due to model error $f_{e}\left(\cdot \right)$. Using the deviation to correct the state covariance described by Equation (9) yields:

(25) $${\bar{P}}_{k}^{*}=\overset{2N}{\underset{i=1}{\sum }}w_{i}^{c}\left({\bar{x}}_{k}^{\left(i\right)}-{\bar{x}}_{k}\right){\left({\bar{x}}_{k}^{\left(i\right)}-{\bar{x}}_{k}\right)}^{T}+Q_{k}+{\overset{ˇ}{P}}_{k}$$

where ${\bar{P}}_{k}^{*}$ denotes the corrected predicted state covariance.

Equation (25) can be further written as:

(26) $$\begin{array}{l} {\bar{P}}_{k}^{*}=\gamma_{k}\left(\overset{2N}{\underset{i=1}{\sum }}w_{i}^{c}\left({\bar{x}}_{k}^{\left(i\right)}-{\bar{x}}_{k}\right){\left({\bar{x}}_{k}^{\left(i\right)}-{\bar{x}}_{k}\right)}^{T}+Q_{k}\right)\\ =\gamma_{k}{\bar{P}}_{k} \end{array}$$

where $\gamma_{k}$ is called the scaling factor, which is defined as:

(27) $$\gamma_{k}I=I+\frac{{\overset{ˇ}{P}}_{k}}{\sum_{i=1}^{2N}w_{i}^{c}\left({\bar{x}}_{k}^{\left(i\right)}-{\bar{x}}_{k}\right){\left({\bar{x}}_{k}^{\left(i\right)}-{\bar{x}}_{k}\right)}^{T}+Q_{k}}$$

where I is a unit matrix.

If we know the deviation ${\overset{ˇ}{P}}_{k}$ in Equation (27) we can determine the scaling factor and calculate the corrected predicted state covariance ${\bar{P}}_{k}^{*}$ directly. However, the deviation ${\overset{ˇ}{P}}_{k}$ is defined by the state prediction error ${\overset{ˇ}{x}}_{k}={\bar{x}}_{k}-x_{k}$. Since the true state $x_{k}$ is generally unknown, it is difficult to directly calculate the deviation ${\overset{ˇ}{P}}_{k}$. In order to solve this problem, this paper extracts all useful information in the innovation sequence via the orthogonality principle [26] to determine the scaling factor.

**Theorem** **1.** *Under the condition of innovation orthogonality, i.e.:*

(28) $$B_{j,k}=E\left[Z_{k}^{I}{}^{T}\cdot Z_{k+j}^{I}\right]=0,\;j=1,2,\cdots .$$

$\gamma_{k}$*can be determined as:*

(29) $$\gamma_{k}=\frac{tr\left(\sum_{j=1}^{M}v_{j}Z_{k-j}^{I}Z_{k-j}^{I}{}^{T}\right)-tr\left(R_{k}\right)}{tr\left(H_{k}{\bar{P}}_{k}H_{k}{}^{T}\right)}$$

*where*$H_{k}$*=*${\frac{\partial h\left(x\right)}{\partial x}|}_{x={\bar{x}}_{k}}$, tr(·)*denotes the trace of a matrix, M is the window size, and*$v_{j}$*is the random weighting factor which meets the condition*$\overset{m}{\underset{j=1}{\sum }}v_{j}=1$.

**Proof.**  Define the estimation error as:

(30) $${\hat{e}}_{k}=x_{k}-{\hat{x}}_{k}.$$

Define the prediction error as:

(31) $${\bar{e}}_{k}=x_{k}-{\bar{x}}_{k}.$$

Substituting Equations (3) and (8) into Equation (31) and expanding f(·) by a Taylor series about ${\hat{x}}_{k-1}$, the prediction error becomes:

(32) $${\bar{e}}_{k}=F_{k}\;{\hat{e}}_{k-1}+q_{k}$$

where $F_{k}$ = ${\frac{\partial f\left(x\right)}{\partial x}|}_{x={\hat{x}}_{k-1}}$.

By considering model error $\;f_{e}\left(\cdot \right),$ Equation (32) becomes:

(33) $${\bar{e}}_{k}=\left(F_{k}+F_{k}^{e}\right)\;{\hat{e}}_{k-1}+q_{k}$$

where $F_{k}^{e}$ = ${\frac{\partial \;f_{e}\left(x\right)}{\partial x}|}_{x={\hat{x}}_{k-1}}$.

Similar to Equation(32), innovation vector $Z_{k}^{I}$ can be represented as:

(34) $$Z_{k}^{I}=H_{k}\;{\bar{e}}_{k}+r_{k}$$

where $H_{k}$ = ${\frac{\partial h\left(x\right)}{\partial x}|}_{x={\bar{x}}_{k}}$.

${\bar{P}}_{k}$ is defined by:

(35) $${\bar{P}}_{k}=E\;\left[\left(x_{k}-{\bar{x}}_{k}\right){\left(x_{k}-{\bar{x}}_{k}\right)}^{T}\right].$$

$P_{{\bar{x}}_{k}{\bar{y}}_{k}}$ is defined by:

(36) $$P_{{\bar{x}}_{k}{\bar{y}}_{k}}=E\;\left[\left(x_{k}-{\bar{x}}_{k}\right){\left(y_{k}-\;{\bar{y}}_{k}\right)}^{T}\right]=E\;\left[\left(x_{k}-{\bar{x}}_{k}\right)Z_{k}^{I}{}^{T}\right].$$

Substituting (34) into (36) and considering (35) and $E\left(r_{k}\right)=0$, we have:

(37) $$\begin{array}{l} P_{{\bar{x}}_{k}{\bar{y}}_{k}}=E\;\left[\left(x_{k}-{\bar{x}}_{k}\right){\left(H_{k}\left(x_{k}-{\bar{x}}_{k}\right)+r_{k}\right)}^{T}\right]\\ =E\;\left[\left(x_{k}-{\bar{x}}_{k}\right){\left(x_{k}-{\bar{x}}_{k}\right)}^{T}H_{k}{}^{T}+r_{k}{}^{T}\right]\\ =\;{\bar{P}}_{k}H_{k}{}^{T}. \end{array}$$

Substituting (33) into (34) yields:

(38) $$Z_{k}^{I}=H_{k}\left[\left(F_{k}+F_{k}^{e}\right){\hat{e}}_{k-1}+q_{k}\right]+r_{k}.$$

Substituting (38) into (28) leads to:

(39) $$\begin{array}{l} B_{j,k}=E\left\{\left[H_{k+j}\left(\left(F_{k+j}+F_{k+j}^{e}\right){\hat{e}}_{k+j-1}+q_{k+j}\right)+r_{k+j}\right]\;\times {\left[H_{k}\left(\left(F_{k}+F_{k}^{e}\right){\hat{e}}_{k-1}+q_{k}\right)+r_{k}\right]}^{T}\right\}\\ =E\left\{\left[H_{k+j}\left(F_{k+j}+F_{k+j}^{e}\right)\left(x_{k+j-1}-{\bar{x}}_{k+j-1}-K_{k+j-1}\left(y_{k+j-1}-\;{\bar{y}}_{k+j-1}\right)\right)\right]\;\times {\left[H_{k}\left(\left(F_{k}+F_{k}^{e}\right){\hat{e}}_{k-1}+q_{k}\right)+r_{k}\right]}^{T}\right\}\\ =E\left\{\left[H_{k+j}\left(F_{k+j}+F_{k+j}^{e}\right)\left(\left(F_{k+j-1}+F_{k+j-1}^{e}\right)\;{\hat{e}}_{k+j-2}-K_{k+j-1}\left(H_{k}\left[\left(F_{k}+F_{k}^{e}\right){\hat{e}}_{k+j-2}\right]\right)\right)\right]\;\times {\left[H_{k}\left(\left(F_{k}+F_{k}^{e}\right){\hat{e}}_{k-1}+q_{k}\right)+r_{k}\right]}^{T}\right\}\\ =E\left\{\left[H_{k+j}\left(F_{k+j}+F_{k+j}^{e}\right)\left(I-K_{k+j-1}H_{k}\left(F_{k}+F_{k}^{e}\right){\hat{e}}_{k+j-2}\right)\right]\;\times {\left[H_{k}\left(\left(F_{k}+F_{k}^{e}\right){\hat{e}}_{k-1}+q_{k}\right)+r_{k}\right]}^{T}\right\}\\ =H_{k+j}\left(F_{k+j}+F_{k+j}^{e}\right)\times \left(\overset{k+j-1}{\underset{i=k+1}{\prod }}\left(I-K_{i}H_{i}\right)\left(F_{i}+F_{i}^{e}\right)\right)\times \left({\bar{P}}_{{\bar{x}}_{k}{\bar{y}}_{k}}-K_{k}B_{0,k}\right) \end{array}$$

where the system and measurement noise covariances are Gaussian white noises, i.e., $\left(r_{i}r_{j}{}^{T}\right)=0,$ $E\left(q_{i}q_{j}{}^{T}\right)=0$ and $E\left(q_{i}r_{j}{}^{T}\right)=0\;\left(i\neq j\right)$, and $B_{0,k}$ is the covariance of innovation vector $Z_{k}^{I}$.

To satisfy the condition (28), (39) is required to be zero, leading to:

(40) $${\bar{P}}_{{\bar{x}}_{k}{\bar{y}}_{k}}-K_{k}B_{0,k}=0.$$

By Taylor series, the predicted measurement covariance given by (11) can be further written as:

(41) $$\begin{array}{l} P_{{\bar{y}}_{k}}=\overset{2N}{\underset{i=1}{\sum }}w_{i}^{c}\left({\bar{y}}_{k}^{\left(i\right)}-{\bar{y}}_{k}\right){\left({\bar{y}}_{k}^{\left(i\right)}-{\bar{y}}_{k}\right)}^{T}+R_{k}\\ =H_{k}{\bar{P}}_{k}H_{k}{}^{T}+R_{k}. \end{array}$$

Replacing ${\bar{P}}_{k}$ with the corrected state covariance ${\bar{P}}_{k}^{*}$ in (26) yields:

(42) $$\begin{array}{l} P_{{\bar{y}}_{k}}^{*}=\left[H_{k}\gamma_{k}{\bar{P}}_{k}H_{k}^{T}\right]+R_{k}\\ =\gamma_{k}\left[H_{k}{\bar{P}}_{k}H_{k}{}^{T}\right]+R_{k} \end{array}$$

where $P_{{\bar{y}}_{k}}^{*}$ denotes the corrected predicted measurement covariance.

Replacing ${\bar{P}}_{k}$ with the corrected state covariance ${\bar{P}}_{k}^{*}$ in (37) yields:

(43) $$P_{{\bar{x}}_{k}{\bar{y}}_{k}}=\gamma_{k}{\bar{P}}_{k}H_{k}{}^{T}.$$

Substituting (42) and (43) into (40), we have:

(44) $$\gamma_{k}\left[H_{k}{\bar{P}}_{k}H_{k}{}^{T}\right]=B_{0,k}-R_{k}.$$

To determine $\gamma_{k}$, we need to know innovation covariance $B_{0,k}$ of innovation vector $Z_{k}^{I}$, which is defined by:

(45) $$B_{0,k}=E\left(Z_{k}^{I}\;Z_{k}^{I}{}^{T}\right).$$

Consider a time window of width *M*, i.e., there are *M* time points $t_{k-1},\;t_{k-2},\;\ldots ,\;t_{k-M}$ in the time window. Thus, (45) can be further written as:

(46) $$B_{0,k}=\frac{1}{M}\overset{M}{\underset{j=1}{\sum }}Z_{k-j}^{I}Z_{k-j}^{I}{}^{T}.$$

Applying the random weighting concept [21] to (46), the random weighting estimation of $B_{0,k}$ can be obtained as:

(47) $$B_{0,k}=\overset{M}{\underset{j=1}{\sum }}v_{j}Z_{k-j}^{I}Z_{k-j}^{I}{}^{T}.$$

where $v_{j}$ is the random weighting factor which meets the condition $\sum_{j=1}^{M}v_{j}=1$.

Substituting (47) into (44) yields:

(48) $$\gamma_{k}\left[H_{k}{\bar{P}}_{k}H_{k}{}^{T}\right]=\overset{M}{\underset{j=1}{\sum }}v_{j}Z_{k-j}^{I}Z_{k-j}^{I}{}^{T}-R_{k}$$

Thus, $\gamma_{k}$ is determined as:

(49) $$\gamma_{k}=\frac{tr\left(\sum_{j=1}^{M}v_{j}Z_{k-j}^{I}Z_{k-j}^{I}{}^{T}\right)-tr\left(R_{k}\right)}{tr\left(H_{k}{\bar{P}}_{k}H_{k}{}^{T}\right)}.$$

The proof of Theorem 1 is completed. □

According to Theorem 1, the corrected predicted state covariance can be calculated as:

(50) $${\bar{P}}_{k}^{*}=\left(\frac{tr\left(\sum_{j=1}^{M}v_{j}Z_{k-j}^{I}Z_{k-j}^{I}{}^{T}\right)-tr\left(R_{k}\right)}{tr\left(H_{k}{\bar{P}}_{k}H_{k}{}^{T}\right)}\right){\bar{P}}_{k}.$$

From the above, we can see that the proposed RWSTUKF takes into account model error by correcting the predicted state covariance. Further, the determination process of the scaling factor $\gamma_{k}$ does not involve the calculation of Jacobian matrix.

### 4.2. Identification of Model Error

Mahalanobis distance is a popular means to detect an uncertain condition [27,28,29]. In this paper, the concept of Mahalanobis distance is adopted to identify contact model error. The Mahalanobis distance $\theta_{k}$ is defined by the innovation and predicted measurement covariance as:

(51) $$\theta_{k}=Z_{k}^{I}{}^{T}{\bar{P}}_{{\bar{y}}_{k}}^{-1}Z_{k}^{I}.$$

$\theta_{k}$ is calculated during the standard UKF procedure to identify contact model error. A contact model error is identified according to the following conditions:

(52) $$\{\begin{array}{l} if\;\theta_{k}\leq \theta_{T}\;Inexistence\;of\;model\;error\\ if\;\theta_{k}>\theta_{T}\;Existence\;of\;model\;error \end{array}$$

where $\theta_{T}$ denotes the predefined threshold value.

### 4.3. Algorithm

**Figure 1:**
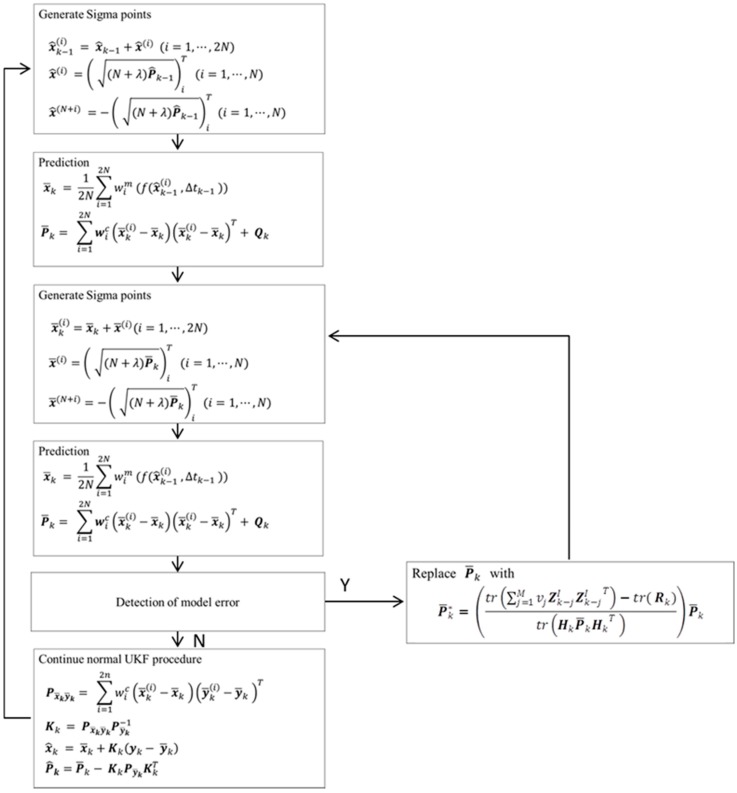
Algorithm of the proposed method.

The detailed procedure of the proposed method is illustrated in Figure 1. In the absence of model error, the proposed method just follows the standard UKF procedure. In the presence of model error, the predicted state covariance is corrected to compensate contact model error and further re-estimate the system state. It can be seen from Figure 1, the proposed method only repeats the process of measurement update (i.e., Step 3), thus maintaining the computational efficiency.

## 5. Performance Evaluation and Discussions

Simulations and experiments were conducted to comprehensively evaluate the proposed method (i.e., RWSTUKF). Simulation analysis was conducted to evaluate the performance of RWSTUKF under three different kinds of contact model error, i.e., initial state estimation error, model simplification error, and local modelling error. Monte Carlo simulations were carried out 100 times. A master-slave robotic indentation system was also developed to conduct experiments for the performance evaluation. In both simulation and experimental analyses, the input force signals are generated based on the nonlinear H-C contact model. The contact forces are reconstructed from the estimated parameters of the nonlinear H-C model and further compared with the input forces as reference to calculate the estimation error. Comparison analysis of the proposed RWSTUKF with the conventional UKF [17] is also discussed in this section.

### 5.1. Initial State Estimation Error

Consider the nonlinear H-C contact model described by (1) with the following constant parameters:

(53) $$K_{k}=10,\;B_{k}=1,\;n_{k}=2,\;p_{k}=1.05$$

by which the input forces are generated from continuously increased displacements. In order to simulate the initial state error, the initial values of the H-C model parameters are set to:

(54) $$K_{0}=150,\;B_{0}=2,\;n_{0}=1,\;p_{0}=1.$$

By comparing (54) with (53), it is obvious that the initial value of state estimation involves a large error.

Trials were conducted by both UKF and RWSTUKF under the same conditions to analyse the effect of the initial state estimation error. The displacement velocity was set to $\dot{d}(t_{k})=$ 0.1 mm, and the window size *m* = 4. $Q_{k}$ was set to $diag{\left(0.01\;mN\right)}_{7\times 7}$, and $R_{k}$ $diag{\left(0.01mN\right)}_{2\times 2}$.

**Figure 2:**
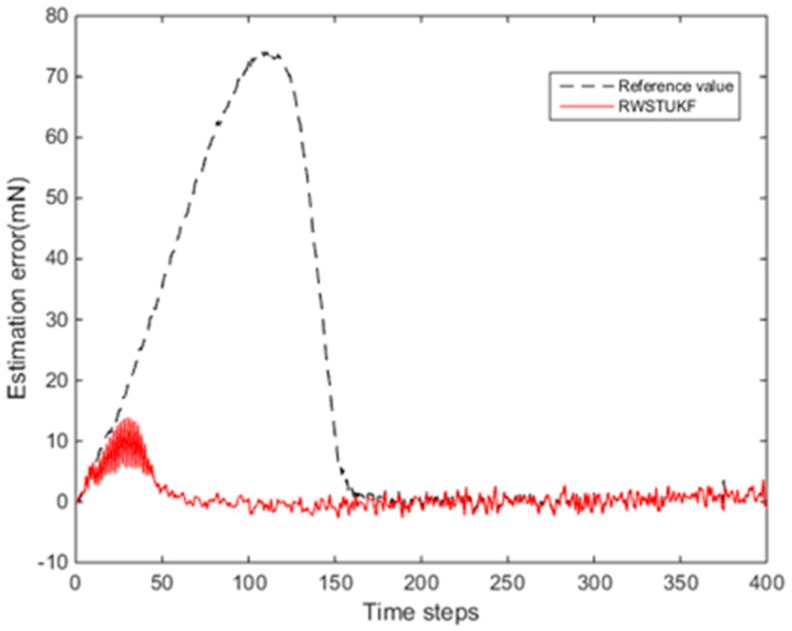
Estimation errors by both unscented Kalman filter (UKF) and random weighting strong tracking unscented Kalman filter (RWSTUKF) under the initial state estimation error.

Figure 2 shows the estimation errors by both UKF and RWSTUKF under the initial state estimation error. Due to the influence of the initial error, the estimation error by UKF is rapidly increased and then converged after 150 time steps, leading to the maximum estimation error of 74.265 mN. However, the estimation error by RWSTUKF is converged only after 50 time steps, showing that the convergence speed of RWSTUKF is three times faster than that of UKF. The resultant maximum estimation error by RWSTUKF is 13.887 mN, which is about six times smaller than that by UKF. This is because RWSTUKF can dynamically adjust predicted state covariance to restrain the disturbance of the initial error on the filtering solution, leading to the improved estimation accuracy than UKF. Table 1 summarizes the estimation errors by both UKF and RWSTUKF. The mean error and RMSE (root mean square error) are 1.8092 mN and 2.9133 mN for RWSTUKF, whereas they are 2.9133 mN and 30.2395 mN for UKF.

### 5.2. Model Simplification Error

Consider the error of model simplification. By letting the parameter *p* = 1, the nonlinear H-C contact model given by (1) is simplified as:

(55) $$F=Kd^{n}+Bd^{n}\dot{d}$$

The input forces are still generated according to (1) under the same conditions as the simulation case in Section 5.1 except that the initial parameter values are set to:

(56) $$K_{0}=10,\;B_{0}=1,\;n_{0}=2,\;p_{0}=1.05$$

Trials were conducted by both UKF and RWSTUKF under the same conditions to analyse the effect of the model simplification error. The displacement velocity was $\dot{d}{\left(t\right)}_{k}$ = 0.01, and the window size *m* = 4. $Q_{k}$ was set to $diag{\left(0.1\;mN\right)}_{7\times 7}$ and $R_{k}$ to $diag{\left(0.1mN\right)}_{2\times 2}.$

**Figure 3:**
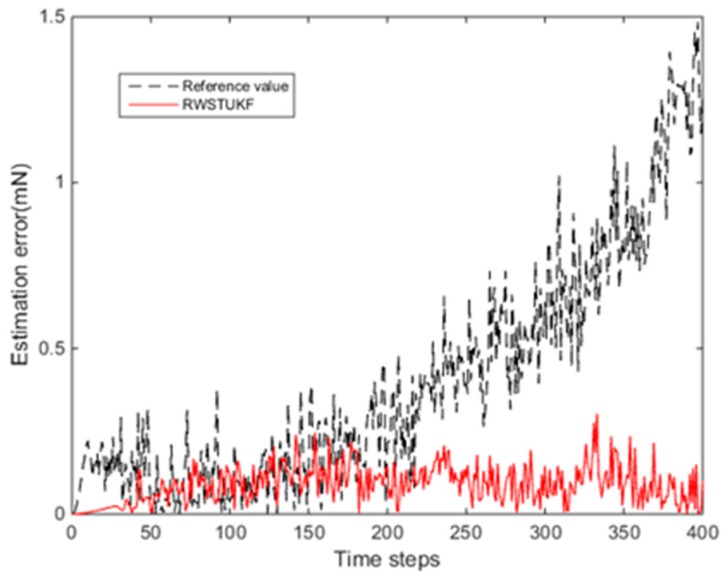
Estimation errors by both UKF and RWSTUKF under the error of model simplification.

Figure 3 shows the estimation errors by both UKF and RWSTUKF under the error of model simplification. The estimation error of UKF is bounded by the maximum of 0.4582 mN within 200 time steps. However, after 200 time steps, due to the disturbance by the error of model simplification, the estimation error of UKF is drastically increased, leading to the maximum error of 1.4844 mN at the end of the test time period. In contrast, within 40 time steps, the estimation error of RWSTUKF is very small, leading to the maximum of 0.0444 mN. After 40 time steps, in spite of a relatively large increase, the estimation error of RWSTUKF is bounded by the maximum of 0.3039 mN, which is still smaller than the maximum error of UKF within 200 time steps. This demonstrates RWSTUKF can restrain the disturbance due to the error of model simplification. Table 2 shows the estimation errors by both UKF and RWSTUKF. The mean error and RMSE are 0.4068 mN and 0.5394 mN for UKF, while they are 0.08977 mN and 0.1063 mN for RWSTUKF. Thus, it is clear that RWSTUKF outperforms UKF.

### 5.3. Local Modelling Error

To simulate local modelling error, a constant prediction error of [0 0 0 0.8 0.8 0 0] is added to the predicted state described by (8) for the time period (200~220 time steps). The other parameters are the same as the simulation case in Section 5.1, except that the initial parameter values are set to be the same as the true values.

**Figure 4:**
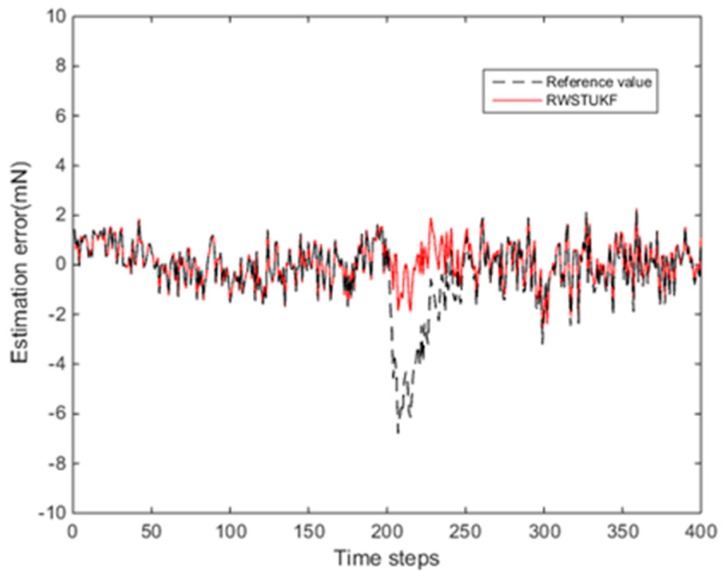
Estimation errors of both UKF and RWSTUKF under local modelling error.

Figure 4 shows the estimation errors by both UKF and RWSTUKF in the presence of the local modelling error. It is clear that the estimation error of UKF is increased dramatically during the time period (200~220 time steps) with the added constant error, leading to the maximum estimation error of 6.7853 mN. In contrast, the estimation error curve of RWSTUKF does not involve a significant change during the entire test time period, especially for the time period with the added constant error. This demonstrates that RWSTUKF can handle the local modelling error. As shown in Table 3, the maximum estimation error is 6.7853 mN for UKF, while it is only 2.588 mN for RWSTUKF, which is about three times smaller than that of UKF. The mean error and RMSE are 0.9531 mN and 1.42 mN for UKF, while they are 0.6911 mN and 0.8590 mN for RWSTUKF.

### 5.4. Robotic Indentation

**Figure 5:**
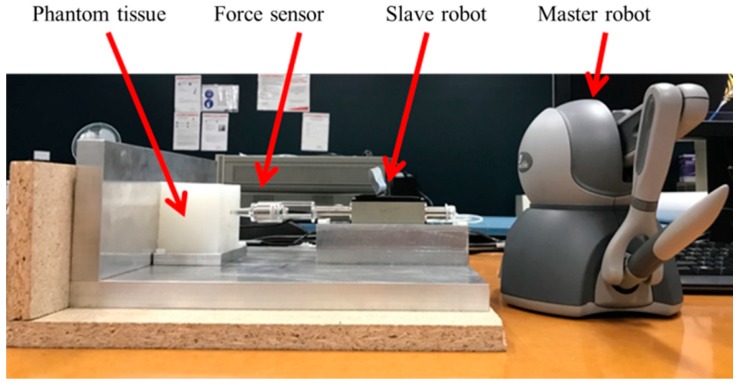
System configuration for robotic indentation.

A master-slave robotic indentation system was developed for the purpose of experimental verification. As shown in Figure 5, the master robot is a Phantom Omni haptic device. The slave robot consists of a linear magnetic motor (LM2070_08011_FMM, FAULHABER) and an indenter of diameter 5 mm, together with a six-axis force sensor (Nano 17 and FTIFPS1) attached between the linear motor and indenter to measure the contact force with the phantom tissue sample. The slave robot can be moved back and forth by manipulating the master robot. With the master-slave robotic system, users can conduct mechanical indentation test on soft material samples to record the contact force and displacement.

A phantom cubic-shape (3 cm × 8 cm × 6 cm) soft tissue was produced from silicone rubber (Ecoflex 0030), which has similar characteristics with human tissues [30]. For the comparison analysis purpose, trials were conducted by both conventional UKF and proposed RWSTUKF under the same conditions. The size of window m was 5, $x_{0}=\left[0.1,\;0.1,\;0.01,\;0.0001,\;0.0001,\;1\;1\right]$, $Q_{k}$ was set to $diag{\left(0.01\;mN\right)}_{7\times 7}$, and $R_{k}$ was set to $diag{\left(0.1mN\right)}_{2\times 2}.$

**Figure 6:**
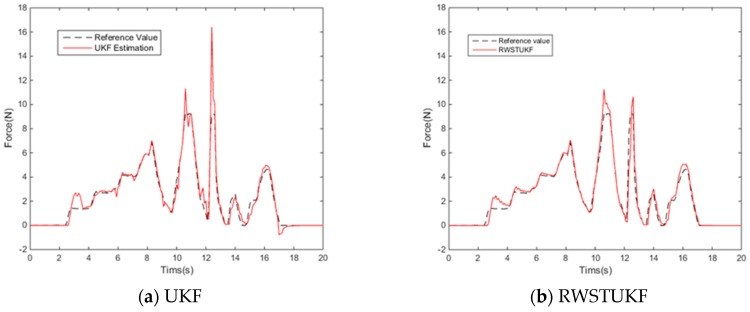
Reconstructed forces via both UKF and RWSTUKF for robotic indentation.

Figure 6 shows the reconstructed forces by both UKF and RWSTUKF with reference to the measured contact force. It can be seen that the UKF estimation involves a large error, leading to the maximum error of 9.6501 N. In contrast, the RWSTUKF estimation follows the reference force curve more closely, leading to the maximum error of 3.3760 N, which is almost three times smaller than that of UKF. This is because RWSTUKF has the capability to handle system model error by online correction of predicted state covariance. Table 4 lists the estimation errors of UKF and RWSTUKF. It can be seen that in addition to the maximum error, the mean error and RMSE of RWSTUKF are also much smaller than those of UKF.

## 6. Conclusions

This paper presents a new method based on the nonlinear H-C contact model for nonlinear soft tissue characterization in the presence of model error. This method identifies model error using the Mahalanobis distance and further incorporates a dynamic scaling factor in predicted state covariance to online compensate identified model error. This scaling factor is determined by combining the principle of innovation orthogonality with the random weighting concept to avoid the cumbersome computation of Jacobian matrix and provide reliable estimation for innovation covariance. The proposed method not only outperforms UKF in the presence of model error, but it also maintains the computational efficiency by correcting predicted state covariance only in the time segments with contact model error. Simulation and experimental results as well as comparison analysis demonstrate that the proposed method can effectively restrain the disturbance of contact model error for online soft tissue characterization.

Future work will focus on the improvement of the proposed method for online soft tissue characterization. It is expected to combine the proposed with artificial intelligence techniques such as genetic algorithms, neural network, pattern recognition and machine learning to automatically determine optimal random weights for the covariance of innovation vector according to the disturbance of abnormal measurements, thus automatically handling contact model error from various sources.

## Figures and Tables

**Table 1:** Estimation errors by both UKF and RWSTUKF under the initial state estimation error.

Errors (mN)	UKF	RWSTUKF
Mean error	16.8818	1.8092
Max error	74.2650	13.8870
RMSE	30.2395	2.9133

**Table 2:** Estimation errors by both UKF and RWSTUKF under the error of model simplification.

Errors (mN)	UKF	RWSTUKF
Mean error	0.4068	0.0897
Max error	1.4844	0.3039
RMSE	0.5394	0.1063

**Table 3:** Estimation errors by both UKF and RWSTUKF under local modelling error.

Errors (mN)	UKF	RWSTUKF
Mean error	0.9531	0.6911
Max error	6.7853	2.5880
RMSE	1.4200	0.8590

**Table 4:** Estimation errors of both UKF and RWSTUKF for robotic indentation.

Errors (N)	UKF	RWSTUKF
Mean error	0.4131	0.2624
Max error	9.6501	3.3760
RMSE	0.9332	0.5088
